# A cross-sectional study: medication safety among cancer in-patients in tertiary care hospitals in KPK, Pakistan

**DOI:** 10.1186/s12913-019-4420-7

**Published:** 2019-08-19

**Authors:** Marium Azim, Ahmad Khan, Tahir Mehmood Khan, Mohammad Kamran

**Affiliations:** 1 Quaid-I-Azam University, QAU Islamabad, Islamabad, Pakistan; 2 Department of Pharmacy, Qauid-I-Azam University, QAU Islamabad, Islamabad, Pakistan; 3 Institute of Pharmaceutical Science, UVAS Lahore, Lahore, Pakistan; 4 Riphah International University Islamabad, Islamabad, Pakistan

**Keywords:** Medication safety, Medication related events, Interventions, Root cause analysis, Contributing factors, Chemotherapy, Potential ADEs

## Abstract

**Background:**

Medication safety in cancer patients receiving complex medication regimens is an important problem in various settings. Medication related events, interceptions and interventions are not well described in this area. We intended to study incidence, types, settings and stages involved, root cause analysis, medication classes involved and the level of harm cause by medication errors in two hospitals providing oncology services comparatively. The severity of incidents and interventions are studied.

**Methods:**

It was a prospective cross sectional study among cancer in-patients of two tertiary care hospitals of KPK. Scale by NCC-MERP was used for evaluation of all medication related incidents. The data obtained was analyzed by IBM SPSS statistics 22 with 95% confidence interval and used the same for other descriptive statistics.

**Results:**

All medication orders were reviewed at both sites (Computerized Prescription Order Entry and HWP systems). Potential ADEs incidence was found high at site 2 (97.5%) while medication errors without harm was high at site 1 (97.5%). Most events occur at prescribing level 87.6 and 81.7% at both sites 1 and 2. Types highly reported involved improper dose 31.4 and 15.5%, monitoring error 14.6 and 15.2% at site 1 and 2. Medications involved in these incidents were antibiotics 44 and 12.7%, antiemetic 7.5 and 15.8% and antineoplastic 2.9 and 9.4% at site 1 and 2. Severity of 3.6 and 36.5% incidents had potential to cause harm at site 1 and 2. Root causes were human factors 62.6 and 72.3%, drug selection 33.6 and 38.8%, and dose selection 39.6 and 15.3% at sites 1 and 2. Contributing factors including staff training 33.6 and 24.3%, system for covering patient care 14.9 and 36.6%, communication system 2.4 and 20.3%, interruptions 9.7 and 7.3% and others 78.8 and 68.6% were highly reported. Preventability of medication errors was 99% at both sites. Intervention was taken in 90.5% events at site 1 (CPOE system) while the incidence lowest at site 2 (HWP system).

**Conclusion:**

Medication related events are high among cancer in-patients at the site lacking updated electronic system for medication prescribing. Proper training about medication safety, reporting and interventions are required.

## Background

According to the Latin phrase “*Premium non nocere*” or “first, do no harm” is one of the main part of the oath the physicians take during practicing medicine. It is because of the complex and human nature of medicine management [1]. But one of the serious challenges to modern health care system is the occurrence of medication errors (MEs). MEs may cause patient harm, increased healthcare cost and ineffective use of health care resources [1–4]. These may also result in increased in-patient hospital days and risk of morbidity and mortality [5]. Patient safety improvement [5] became a global concern for all including healthcare professionals (HCPs), policy makers and the public [6]. The medication use process is highly prone to errors [5]. MEs among prescriptions and intravenous administration of drugs to the patients were highly reported [7–9]. The severity ranged from minor harm to life threatening incidents [1].

Attitudes like hiding errors or punitive approach causes difficulty in creating safe culture and patient safety among HCPs [1]. A change in culture approach greatly reduced error incidence from 11.4 to 7.3% [1]. 59% errors had ability to cause harm, 25% actually caused harm while 30% were harmless during an academic study. The highest rate was reported in intensive care patients (21.1 per 100 admissions) during ordering (96%) [6]. In 16-24% of events MEs occurred either in cluster of sequence or in the form of groups. Highest rate was reported during ordering (32%) and administration (39%) [10].

Cancer patients are more prone to medication errors like prescription errors with over dosage. Anti-neoplastic medicines have several toxic effects at their therapeutic doses as well because of the narrow therapeutic index, complex regimes and the vulnerability of cancer patients to potential harms [2, 11–13]. All these factors make anti-neoplastic drugs the second most common cause of death. This fact makes it important to well recognize the predictors involved in medication errors [12]. Studies showed > 5% of chemotherapy orders contained at least one ME [12, 13], > 50% of administration errors [14], > 40% omission errors, > 20% errors due to brand names and due to abbreviations used for chemotherapy and pre-medications [11].

In this study we tried to evaluate the incidence of medication errors among cancer inpatients along with humanistic and system related causes and contributing factors. The medication errors were evaluated according to the scale established by National Coordinating Center for Medication Errors Reporting and Preventions (NCC-MERP).

## Methods

### Study design

The study was approved by the scientific committee of the institute and both hospital sites. The study design was cross-sectional and extended over the period of nine months at both sites. The medical records were directly observed by the principal investigator for all in-patients receiving chemotherapy.

### Study sites

Both study sites were non-government health institutes. The pharmacy services at both institutes differed on the basis of system operation. Site 1 used highly upgraded software with computer order entry system (CPOE)/computer decisions supportsystem (CDSS). While at Site 2 all medication orders were handwritten by the oncologists. The medication orders were forwarded by nurses through software to in-patient pharmacies while dispensed by pharmacists.

### Data collection tool

Medication orders were reviewed after prescription by the principle investigator. Data collection tool was designed by the criteria of NCC-MERP. While medication orders were reviewed with several approved medical guidelines and literature such as Medscape, British National Formulary (BNF), National Comprehensive Cancer Network (NCCN) guidelines and other drug indices which were followed at the settings., More than one type, stage, causes and contributing factors were included for a medication order where needed. Where a number of causes and contributing factors lead to the occurrence of incident, these were reported separately.

### Eligibility criteria

Medication orders of in-patients (both adults and pediatrics) with breast, ovarian, prostrate, blood and gastric cancer receiving chemotherapy and/or supportive care medications were reviewed. Patients receiving radiotherapy or anti-neoplastic drugs for indications other than cancer were excluded. Patients visiting multiple times for chemotherapy were included again.

### Data analysis

We calculated the rate of incidents per 1000 medication orders. Mean with 95% confidence interval was obtained for the types, causes and contributing factors of incidents along with rate and a two-tailed significance via IBM SPSS statistics 22. Further descriptive analysis for the stages and medication classes involved in medication related events.

## Results

In this study, 75 charts (in CPOE system) with 2925 medication orders at site 1 and 76 charts (in HWP system) with 1657 medication orders at site 2 were reviewed. A total of 1195 incidents were found during review of all medication orders (*n* = 4852). A rate of 0.25 incidents per 1000 orders occurred.

### Types of incidents at both sites

At site 1 (CPOE system) approximately 185 incidents (31.4%, CI 0.28-0.35, *P* = 0.000) showed improper dose, 133 incidents (22.6%, CI 0.19-0.26, *P* = 0.000) of potential problems with the lack of treatment effectiveness, 116 incidents (19.7%, CI 0.16-0.23, P = 0.000) of wrong drug, 86 incidents (14.6%, CI 0.12-0.17, *P* = 0.000) of monitoring errors including contraindications and drug interactions, 69 incidents (11.7%, CI 0.09-0.14, *P* = 0.000) of wrong time or schedule of drug administration and 229 other types of incidents (38.9%, CI 0.35-0.43, *P* = 0.000) such as not highlighting administration considerations in 33 incidents (5.6%, CI 0.04-0.07, *P* = 0.000), wrong indication for drug in 61 incidents (10.4%, CI 0.08-0.13, *P* = 0.000) and missing information about the drugs in 18 incidents (3.1%, CI 0.02-0.04, *P* = 0.000) (Table 1).

**Table 1:** Types of incidents occurring among oncology patients

Type of incidents	Site 1 (CPOE)						Site 2 (HWP)						Total
	No.	%	Mean	95% CI		Sig. (2-tailed)	No.	%	Mean	95% CI		Sig. (2-tailed)	
				Lower	Upper					Lower	Upper		
Dose Omission	5	0.8	0.01	0.00	0.02	0.025	24	4	0.04	0.02	0.05	0.000	29
Wrong Strength or concentration	17	2.9	0.03	0.02	0.04	0.000	18	3	0.03	0.02	0.05	0.000	35
Wrong Dosage form	42	7.1	0.07	0.05	0.09	0.000	25	4.1	0.04	0.03	0.06	0.000	67
Wrong route of administration	36	6.1	0.06	0.04	0.08	0.000	4	0.7	0.01	0	0.01	0.000	40
Wrong time or schedule	69	11.7	0.12	0.09	0.14	0.000	52	8.6	0.09	0.07	0.11	0.000	121
Wrong Rate (IV bolus/injection/infusion)	14	2.4	0.02	0.01	0.04	0.000	28	4.6	0.05	0.03	0.07	0.000	42
Wrong Drug	116	19.7	0.20	0.16	0.23	0.000	37	6.1	0.06	0.04	0.08	0.000	153
Wrong Technique	19	3.2	0.03	0.02	0.05	0.000	48	7.9	0.08	0.06	0.1	0.000	67
Wrong Duration	1	0.2	0.00	0.00	0.01	0.318	17	2.8	0.03	0.02	0.04	0.000	18
Wrong Patient	1	0.2	0.00	0.00	0.01	0.318	–	–	–	–	–	–	1
Improper Dose	185	31.4	0.31	0.28	0.35	0.000	94	15.5	0.16	0.13	0.19	0.000	279
Inappropriate diluents	1	0.2	0.00	0.00	0.01	0.318	10	1.7	0.02	0.01	0.03	0.002	11
Inappropriate Labeling	12	2	0.02	0.01	0.03	0.001	51	8.4	0.09	0.07	0.11	0.000	63
Monitoring Error (Including CIs*^a^ and DIs*^b^)	86	14.6	0.15	0.12	0.17	0.000	92	15.2	0.14	0.12	0.17	0.000	178
Deteriorated Drug Error	7	1.2	0.01	0.00	0.02	0.008	1	0.2	0	0	0.01	0.318	8
Potential problem with the lack of treatment effectiveness	133	22.6	0.23	0.19	0.26	0.000	53	8.7	0.08	0.06	0.1	0.000	186
Patient suffers or will possibly suffer from ADEs	69	11.7	0.12	0.09	0.14	0.000	87	14.4	0.15	0.12	0.18	0.000	156
Drug treatment more costly than necessary	52	8.8	0.09	0.07	0.11	0.000	109	18	0.18	0.14	0.21	0.000	161
Other type of incidents	229	38.9	0.39	0.35	0.43	0.000	345	56.9	0.69	0.6	0.78	0.000	574
Other types (breakup)													
Unauthorized medication	12	2	0.02	0.01	0.03	0.001	13	2.1	0.02	0.01	0.03	0.000	25
Replacing medication without approval	6	1	0.01	0.00	0.02	0.014	1	0.2	0	0	0.01	0.318	7
Requesting more than required according to order	15	2.5	0.03	0.01	0.04	0.000	12	2	0.02	0.01	0.03	0.001	27
No entry of drug administration record	–	–	–	–	–	–	14	2.3	0.02	0.01	0.04	0.000	14
Forgetting to prepare medication	1	0.2	0.00	0.00	0.01	0.318	20	3.3	0.03	0.02	0.05	0.000	21
Inappropriate storage							6	1	0.01	0	0.02	0.014	6
Not highlighting administration considerations	33	5.6	0.00	0.04	0.07	0.000	16	2.6	0.02	0.01	0.04	0.000	49
No entry of patient indication	–	–	–	–	–	–	7	1.2	0.01	0	0.02	0.008	7
Missing necessary information	18	3.1	0.00	0.02	0.04	0.000	107	17.7	0.17	0.14	0.2	0.000	125
Unnecessary or medication without need	11	1.9	0.06	0.01	0.03	0.001	68	11.2	0.11	0.08	0.13	0.000	79
Medication non-compliance	–	–	–	–	–	–	2	0.3	0	0	0.01	0.157	2
Use of abbreviations	–	–	–	–	–	–	5	0.8	0.01	0	0.01	0.025	5
Product not available	20	3.4	0.00	0.02	0.05	0.000	24	4	0.04	0.02	0.06	0.000	44
No drug given for indication	51	8.7	0.03	0.06	0.11	0.000	33	5.4	0.06	0.04	0.08	0.000	84
Therapeutic duplication	37	6.3	0.02	0.04	0.08	0.000	20	3.3	0.03	0.02	0.05	0.000	57
Entry of not administered dose in MAR*^c^	1	0.2	0.00	0.00	0.01	0.318	3	0.5	0	0	0.01	0.083	4
Incorrect entry in MAR*^c^	1	0.2	0.00	0.00	0.01	0.318	5	0.8	0.01	0	0.02	0.025	6
Wrong Indication for drug	61	10.4	0.03	0.08	0.13	0.000	34	5	0.06	0.04	0.08	0.000	95
Incorrect IV*^d^ to PO*^e^ conversion	1	0.2	0.09	0.00	0.01	0.318	9	1.5	0.02	0.01	0.03	0.003	10
Total	1362	–	–	–	–	–	1494	–	–	–	–	–	2856

C.Is*^a^ Contraindicated, DIs*^b^ Drug interactions, MAR*^c^ Medication Administration Record, IV*^d^ intravenous and PO*^e^ Per Oral

At site 2 (HWP system) approximately 94 incidents (15.5%, CI 0.13-0.19, *P* = 0.000) showed improper dose, 92 incidents (15.2%, CI 0.12-0.17, *P* = 0.000) of monitoring errors including contraindications and drug interactions, 52 incidents (8.6%, CI 0.07-0.11, *P* = 0.000) of wrong time or schedule of drug administration, 51 incidents (8.4%, CI 0.07-0.11, *P* = 0.000) of no or improper labeling, 53 incidents (8.7%, CI 0.06-0.1, *P* = 0.000) of potential problems with the lack of treatment effectiveness, and 345 other types of incidents (56.9%, CI 0.6-0.78, P = 0.000) such as missing information about the drugs in 107 incidents (17.7%, CI 0.14-0.2, P = 0.000), 68 incidents (11.2%, CI 0.08-0.13, P = 0.000) of unnecessary medications including many others including product unavailability, medications without need and no entry in records (Table 1).

### Causes leading towards occurrence of incidents

Among many causes leading towards incidence occurrence the highly reported cause/s at site 1 (CPOE system) were dose or dosage selection in 233 incidents (39.6%, CI 0.36-0.44, 0.000), drug selection in 198 incidents (33.6%, CI 0.3-0.37, 0.000), human factors in 369 incidents (62.6%, CI 0.59-0.63, *P* = 0.000) which further includes performance deficit of staff in 137 incidents (23.3%, CI 0.20-0.27, P = 0.000), Dosage and rate miscalculations in 108 incidents (18.3%, CI 0.15-0.21, *P* = 0.000), transcribing error in 26 incidents (4.4%, CI 0.03-0.06, *P* = 0.000) and confrontational behavior of the staff in 43 incidents (7.3%, CI 0.05-0.09, P = 0.000). Other causes included lack of monitoring and cross checking practices in 61 incidents (10.4%, CI 0.08-0.13, P = 0.000) and prescribing unnecessary medicines in 30 incidents (5.1%, CI 0.03-0.07, *P* = 0.000) (Table 2).

**Table 2:** Causes leading towards occurrence of incidents

Causes of incidents	Site 1 (CPOE)						Site 2 (HWP)						Total
	No.	%	Mean	95% CI		Sig. (2-tailed)	No.	%	Mean	95% CI		Sig. (2-tailed)	
				Lower	Upper					Lower	Upper		
Communication	21	3.5	0.04	0.02	0.06	0.000	58	9.7	0.19	0.14	0.24	0.000	79
Name and Sound confusion	–	–	–	–	–	–	1	0.2	0.00	0.00	0.01	0.318	1
Labeling	8	1.4	0.01	0.00	0.02	0.005	28	4.6	0.05	0.03	0.07	0.000	36
Packaging or design	6	1	0.01	0.00	0.02	0.014	–	–	–	–	–	–	6
Drug selection	198	33.6	0.34	0.3	0.37	0.000	235	38.8	0.39	0.35	0.43	0.000	433
Inappropriate drug form	54	9.2	0.09	0.07	0.12	0.000	27	4.5	0.05	0.03	0.06	0.000	81
Dose or dosage selection	233	39.6	0.40	0.36	0.44	0.000	93	15.3	0.15	0.13	0.28	0.000	326
Inappropriate duration of therapy	15	2.5	0.03	0.01	0.04	0.000	27	4.5	0.05	0.03	0.06	0.000	42
DRP*^a^ with drug use in spite of instruction	–	–	–	–	–	–	6	1	0.02	0.00	0.03	0.033	6
DRPs*^a^ related to logistics	2	0.3	0.00	0.00	0.01	0.157	66	10.9	0.10	0.08	0.13	0.000	68
DRPs*^a^ related to the patient personality or behavior	7	1.2	0.00	0.00	0.01	0.008	7	1.2	0.01	0.00	0.02	0.008	14
Human factors (breakup)	369	62.6	0.63	0.59	0.67	0.000	438	72.3	0.72	0.68	0.75	0.000	807
Knowledge deficit	55	9.3	0.09	0.07	0.12	0.000	57	9.4	0.09	0.07	0.11	0.000	112
Performance deficit	137	23.3	0.23	0.20	0.27	0.000	162	26.7	0.29	0.25	0.33	0.000	299
Dosage/rate Miscalculations	108	18.3	0.18	0.15	0.21	0.000	42	6.9	0.08	0.05	0.1	0.000	150
System error	7	1.2	0.01	0.00	0.02	0.008	5	0.8	0.01	0.00	0.02	0.025	12
Error in stocking	–	–	–	–	–	–	28	4.6	0.05	0.03	0.06	0.000	28
Drug preparation or dilution error	5	0.8	0.01	0.00	0.02	0.025	14	2.3	0.02	0.01	0.03	0.000	19
Transcription error	26	4.4	0.04	0.03	0.06	0.000	51	8.4	0.08	0.06	0.1	0.000	77
High Volume workload	40	6.8	0.07	0.05	0.09	0.000	42	6.9	0.07	0.05	0.09	0.000	82
Fatigue or lack of sleep	–	–	–	–	–	–	8	1.3	0.01	0.00	0.02	0.005	8
Confrontational or intimidating behavior	43	7.3	0.07	0.05	0.09	0.000	135	22.3	0.21	0.18	0.25	0.000	178
Others (breakup)	115	19.5	0.20	0.16	0.23	0.000	247	40.8	0.40	0.36	0.44	0.000	362
Lack of monitoring or cross checking practices	61	10.4	0.10	0.08	0.13	0.000	155	25.6	0.25	0.21	0.28	0.000	216
Lack of all-time oncology pharmacist	14	2.4	0.02	0.01	0.04	0.000	44	7.3	0.07	0.05	0.09	0.000	58
Less knowledge of antibiotic stewardship	27	4.6	0.05	0.03	0.06	0.000	18	3	0.03	0.02	0.05	0.000	45
Unnecessary medicine/s	30	5.1	0.05	0.03	0.07	0.000	45	7.4	0.07	0.05	0.1	0.000	75
Wrong drug combination/s	20	3.4	0.03	0.02	0.05	0.000	3	0.5	0.01	0.00	0.01	0.083	23
Total	1601						2042						3643

While at site 2 (HWP system) causes leading highly towards incidence occurrence were drug selection in 235 incidents (38.8%, CI 0.35-0.43, 0.000), dose or dosage selection in 93 incidents (15.3%, CI 0.13-0.28, 0.000), communication barriers caused 58 incidents (9.7%, CI 0.14-0.24, *P* = 0.000), human factors in 438 incidents (72.3%, CI 0.68-0.75, P = 0.000) which further includes performance deficit of staff in 162 incidents (26.7%, CI 0.25-0.33, P = 0.000), confrontational behavior of the staff in 135 incidents (22.3%, CI 0.18-0.25, P = 0.000), transcribing error in 51 incidents (8.4%, CI 0.06-0.1, *P* = 0.000). Among other causes the most common were lack of monitoring practices i.e. 155 incidents (25.6%, CI 0.21-0.28, *P* = 000) (Table 2).

### Factors contributing towards the occurrence of incidents

Among the contributing factors leading towards occurrence of incidents the highly reported factors at site1 (CPOE system) included staff training in 198 incidents (33.6%, CI 0.30-0.38, *P* = 0.000), System covering patient care in 88 incidents (14.9%, CI 0.12-0.18, *P* = 0.000), frequent interruptions in 57 incidents (9.7%, CI 0.07-0.12, P = 0.000) along with other factors in 464 incidents (78.8%, CI 0.75-0.82, P = 0.000). Other factors further included highest rate of prescribing practices in 261 incidents (44.3%, CI 0.40-0.48, *P* = 0.000), lack of updated knowledge in 126 incidents (21.4%, CI 0.18-0.25, *P* = 0.000) and staff attitude in 92 incidents (15.6%, CI 0.14-0.21, *P* = 0.000) as factors which played role in the occurrence of incidents at different levels (Table 3).

**Table 3:** Factors contributing towards the occurrence of incidents and their severity index

	Site 1 (CPOE)						Site 2 (HWP)						
	No.	%	Mean	95% CI		Sig. (2-tailed)	No.	%	Mean	95% CI		Sig. (2-tailed)	Total
				U	L					U	L		
Contributing factors													
Noise level	–	–	–	–	–	–	8	1.3	0.01	0.00	0.02	0.005	8
Staff training	198	33.6	0.341	0.30	0.38	0.000	147	24.3	0.24	0.21	0.28	0.000	345
Staffing	4	0.7	0.007	0.00	0.01	0.045	49	8.1	0.08	0.06	0.10	0.000	53
Patient counseling	19	3.2	0.032	0.02	0.05	0.000	34	5.6	0.05	0.04	0.07	0.000	53
Nursing floor or pharmacy stock	7	1.2	0.012	0.00	0.02	0.008	24	4	0.04	0.02	0.06	0.000	31
Frequent interruptions and distractions	57	9.7	0.097	0.07	0.12	0.000	44	7.3	0.07	0.05	0.09	0.000	101
System covering patient care	88	14.9	0.149	0.12	0.18	0.000	222	36.6	0.36	0.32	0.40	0.000	310
Lack of availability of HCPs*^b^	8	1.4	0.014	0.00	0.02	0.005	35	5.8	0.06	0.04	0.08	0.000	43
Policies and procedures	43	7.3	0.073	0.05	0.09	0.000	61	10.1	0.10	0.08	0.13	0.000	104
Communication systems	14	2.4	0.024	0.01	0.04	0.000	123	20.3	0.21	0.17	0.24	0.000	137
Hand written medication orders	–	–	–	–	–	–	67	11.1	0.11	0.08	0.13	0.000	67
Assignment of inexperienced personnel	7	1.2	0.012	0.00	0.02	0.008	99	16.3	0.16	0.13	0.19	0.000	106
Others (breakup)	464	78.8	0.788	0.75	0.82	0.000	416	68.6	0.68	0.65	0.72	0.000	880
MAR*^c^ maintenance in hard	–	–	–	–	–	–	16	2.6	0.03	0.01	0.04	0.000	16
PMR*^d^ in hard form	–	–	–	–	–	–	2	0.3	0.00	0.00	0.01	0.157	2
Cross checking practice	28	4.8	0.048	0.03	0.06	0.000	57	9.4	0.10	0.07	0.12	0.000	85
Lack of interdisciplinary approach	–	–	–	–	–	–	78	12.9	0.13	0.10	0.16	0.000	78
Task performed by wrong personnel	1	0.2	0.002	0.00	0.01	0.318	10	1.7	0.02	0.01	0.03	0.002	11
Missing documented protocols	–	–	–	–	–	–	3	0.5	0.01	0.00	0.01	0.083	3
Prescribing practices	261	44.3	0.443	0.40	0.48	0.000	248	40.9	0.41	0.37	0.45	0.000	509
Lack or delay of culture sensitivity tests	–	–	–	–	–	–	17	2.8	0.03	0.02	0.05	0.000	17
Staff attitude	92	15.6	0.177	0.14	0.21	0.000	95	15.7	0.16	0.13	0.19	0.000	187
Lack of updated knowledge	126	21.4	0.214	0.18	0.25	0.000	23	3.8	0.04	0.02	0.05	0.000	149
Total	1417	–	–	–	–	–	1878	–	–	–	–	–	3295
Patient outcome according to severity index													
No error/Category A^*e^	14	2.4	0.02	0.01	0.04	0.000	7	1.1	0.00	0.00	0.01	0.157	21
Error, but no harm	554	94.1	1.76	1.07	1.82	0.000	376	62.1	1.51	1.41	1.61	0.000	930
Category B*^f^	146	24.8	–	–	–	–	24	4	–	–	–	–	170
Category C*^g^	334	56.7	–	–	–	–	163	26.9	–	–	–	–	497
Category D*^h^	74	12.6	–	–	–	–	189	31.2	–	–	–	–	263
Error, and caused harm	21	3.6	0.04	0.02	0.05	0.000	221	36.5	0.56	0.50	0.63	0.000	242
Category E*^i^	21	3.6	–	–	–	–	115	19	–	–	–	–	136
Category F*^j^	–	–	–	–	–	–	90	14.9	–	–	–	–	90
Category G*^k^	–	–	–	–	–	–	15	2.5	–	–	–	–	15
Category H*^l^	–	–	–	–	–	–	1	0.2	–	–	–	–	1
Error, and caused death/ Category I*^m^	–	–	–	–	–	–	2	0.3	0.02	0.00	0.04	0.023	2
Total	589	–	–	–	–	–	606	–	–	–	–	–	1195

DRPs*^a^ Drug related problems, HCPs*^b^ Health care professionals, MAR*^c^ Medication administration record, PMR*^d^ Patient medication record. Category A^*e^: Circumstances or events that have the capacity to cause error, Category B*^f^: An error occurred but the error did not reach the patient, Category C*^g^: An error occurred that reached the patient, but did not cause patient harm, Category D*^h^: Error occurred, reached patient, require monitoring to ensure no harm is occurred and/or require intervention to prevent harm, Category E*^I^: Error occurred, resulting or contributing to temporary harm to the patient, requiring intervention, Category F*^j^: Error occurred, contributing to or resulting in temporary harm to the patient, and requiring initial or prolongation of hospitalization, Category G*^k^: Error occurred, contributing to resulting in permanent patient harm, Category H*^l^: Error occurred, requiring intervention to sustain life, and Category I*^m^: Error occurred, resulting in death of the patient

At site 2 (HWP system) the presence of factors like staff training in 147 incidents (24.3%, CI 0.21-0.28, *P* = 0.000), system covering patient care in 222 incidents (36.6%, CI 0.32-0.40, *P* = 0.000), Communication system in 123 incidents (20.3%, CI 0.17-0.24, *P* = 0.000), assignment of inexperienced personnel in 99 incidents (16.3%, CI 0.13-0.19, *P* = 0.000), policies and procedures in 61 incidents (10.1%, CI 0.08-0.13, *P* = 0.000) along with other factors in 416 incidents (68.6%, 0.65-0.72, *P* = 0.000) which lead to the occurrence of these incidents. Among other factors prescribing practices in 248 incidents (40.9%, CI 0.37-0.45, P = 0.000), staff attitude in 95 incidents (15.7%, CI 0.13-0.19, P = 0.000), lack of interdisciplinary approach in 78 incidents (12.9%, CI 0.10-0.16, *P* = 0.000), cross checking practice in 57 incidents (9.4%, CI 0.07-0.12, *P* = 0.000) and lack of updated knowledge in 23 incidents (3.8%, CI 0.02-0.05, P = 0.000) resulted in the occurrence of these incidents (Table 3).

### Patient outcome

Patient outcome analysis according to the severity index (NCC-MERP) showed many incidents caused no harm at both sites with a figure of no harm events at site 1 (CPOE system) of 554 incidents (94%, CI 1.07-1.82, *P* = 0.000) and 376 incidents (62%, CI 1.41-1.61, *P* = 0.000) at site 2 (HWP system). Among no harm events the Category C events were highly reported at site 1 (CPOE system) with an occurrence of 334 incidents (56.7%) and Category D events were highly reported at site 2 (HWP system) with an occurrence of 189 incidents (31.2%).

The events which caused harm were high in occurrence at site 2 (HWP system) with an incidence of 221 (36.5%, CI 0.50-0.63, P = 0.000) and at site 1 (CPOE system) an incidence of 21 (3.6%, CI 0.02-0.05, P = 0.000). The Category E events were reported highly at site 1 (CPOE system) with an occurrence of 21 incidents (3.6%) and 115 incidents (19%) at site 2 (HWP system). While occurrence of Category F events reported were 90 incidents (14.9%), and Category G events reported were 15 incidents (2.5%) only at site 2 (HWP system) (Table 3).

### Settings and stages of incidents occurrence and discovery

At site 1 (CPOE system) setting of initial incident showed greater incidence at the level of ward in 287 incidents (48.7%) and prescribers’ office in 285 incidents (48.4%). Site 2 (HWP system) also showed greater incidence of setting of initial incident at the level of ward in 294 incidents (48.5%) and at prescribers’ office in 269 incidents (44.4%) (Table 4).

**Table 4:** Rate of incidents at different settings and stages; their preventability and interventions

Variable	Site 1 (CPOE)		Site 2 (HWP)		Total	
	N	%	N	%	N	%
Setting of initial incidents						
Oncology ward	287	48.7	294	48.5	581	97.2
Pharmacy	12	2	28	4.6	40	6.6
Prescriber’s Office	285	48.4	269	44.4	554	92.8
Patient home	–	–	10	1.7	10	1.7
Other	5	0.8	5	0.8	10	1.6
Stages of initial error						
Physician	516	87.6	495	81.7	1011	169.3
Consultant	372	63.2	389	64.2	761	127.4
Medical Officer	139	23.8	96	15.8	235	39.6
Fellow	–	–	10	1.7	10	1.7
Resident	5	0.6	–	–	5	0.6
Pharmacist	39	6.6	21	3.5	56	10.1
Prescription receiving or checking	29	4.9	15	2.5	43	7.4
Prescription filling	9	1.5	6	1	12	2.5
Dispensing	1	0.2	–	–	1	0.2
Nurse	25	4.3	75	12.3	100	16.6
Medication received	24	4.1	62	10.2	86	14.3
Administered	1	0.2	13	2.1	14	2.3
Patient Care Giver	14	2.4	5	0.8	19	3.2
Other	2	0.3	4	0.7	6	1
Support personnel	2	0.3	–	–	2	0.3
Health Profession Students	–	–	1	0.2	1	0.2
Physician assistant	–	–	3	0.5	3	0.5
Unknown	–	–	10	1.7	10	1.7
Stage of error discovery						
Physician	48	8.1	24	4	72	12.1
Consultant	29	4.9	–	–	29	4.9
Medical Officer	19	3.2	13	2.1	32	5.3
Fellow	–	–	11	1.8	11	1.8
Resident	–	–	–	–	–	–
Pharmacist	482	81.8	14	2.3	496	84.1
Prescription receiving or checking	378	64.2	11	2.1	389	66.3
Prescription filling	90	15.3	3	0.5	93	15.8
Dispensing	14	2.4	–	–	14	2.4
Nurse	15	2.6	30	5	45	7.6
Medication received	8	1.4	24	4	32	5.4
Administered	7	1.2	6	1	13	2.2
Patient care giver	–	–	1	0.2	1	0.2
Other	44	7.5	536	88.4	580	95.9
Health Profession Students	–	–	9	1.5	9	1.5
Support personnel	1	0.2	–	–	1	0.2
Physician Assistant	1	0.2	–	–	1	0.2
Primary Investigator (PI)	42	7.1	527	86.9	569	94
Unknown	–	–	1	0.2	1	0.2
Rate of MEs^a^ without harm and potential ADEs^b^						
MEs without harm	574	97.5	20	3.3	594	100.8
Potential ADEs	15	2.5	586	96.7	601	99.2
Preventability of the event						
Preventability	582	98.8	601	99.2	1183	198
Not-preventable	7	1.2	5	0.8	12	2
Total	589	100	606	100	1195	
Intervention						
Medical intervention	533	90.5	48	7.9	581	98.4

^a^ Medication errors, ^b^ Adverse drug events

Stages of initial incident occurrence showed higher incidence at the level of physician at incident rate of 516 (87.6%), pharmacist at incident rate of 39 (6.6%), nurse at incident rate of 25 (4.3%), patient care giver at incident rate of 14 (2.4%) and others at incident rate of 2 (0.3%) at site 1 (CPOE system). At site 2 (HWP system) initial error showed higher occurrence at the level of physician at incident rate of 495 (81.7%), pharmacist at incident rate of 21 (3.5%) followed by nurse at incident rate of 75 (12.3%), patient care giver at incident rate of 5 (0.8%) and others at incident rate of 4 (0.7%) (Table 4).

Stages of incident discovery showed a greater percentage of discovery and interception at the level of pharmacy at site 1 (CPOE system) at a rate of 482 incidents (81.8%) followed by physician at a rate of 48 incidents (8.1%), nursing at a rate of 15 incidents (2.6%) and others at a rate of 44 incidents (7.5%). At site 2 ((HWP system) the primary investigator among others (536 incidents, 88.4%) discovered and intercepted greater number of incidents at a rate of 536 incidents (86.9%) followed by nursing at a rate of 30 incidents (5%) and pharmacy at a rate of 14 incidents (2.3%) (Table 4).

### Rate of incidents, preventability and interventions

The medication review process identified incidence rate of 574 (97.5%) medication errors without harm at site 1 (CPOE system) and 20 (3.3%) at site 2 (HWP system) among all reported incidents. The preventability analysis showed a rate of prevention of 582 incidents (98.8%) and 7 incidents (1.2%) were not preventable at site 1 (CPOE system). At site 2 (HWP system) 601 incidents (99.2%) showed preventability and 5 incidents (0.8%) were not preventable.

Medical interventions were done and reported at site 1 (CPOE system) by all HCPs involved with an incidence rate of 533 incidents (90.5%) as compared to site 2 (HWP system) with an incidence rate of 48 incidents (8%) (Table 4).

### Medication classes involved

The medication classes involved in the occurrence of medication related events reported at site 1 (CPOE system) highly reported antibiotics in 259 incidents (44%), analgesics and antipyretics (NSAIDs and Opiates) in 67 incidents (11.4%), and antiemetic in 44 incidents (7.5%) along with other classes including antihistamines, antineoplastic agents, anti-diabetic agents, coagulants and drugs acting on central nervous system. At site 2 (HWP system) the medication classes highly reported included antiemetic in 96 incidents (15.8%), antibiotics in 77 incidents (12.7%), dietary supplements in 72 incidents (11.9%), antineoplastic agents in 57 incidents (9.4%), analgesics and antipyretics (NSAIDs and Opiates) in 55 incidents (9.1%) along with other classes like replacement solutions, human albumin, coagulants and cathartics and laxatives (Table 5).

**Table 5:** Medication classes involved in occurrence of incidents

Medication classes	Site 1 (CPOE)		Site 2 (HWP)		Total	
	N	%	N	%	N	%
Analgesics and antipyretics (NSAIDs*^a^ + Opiates)	67	11.4	55	9.1	122	20.5
Antacids	31	5.3	42	6.9	73	12.2
Antibiotics or antibacterial agents	259	44	77	12.7	336	56.7
Anticonvulsants	1	0.2	1	0.2	2	0.4
Antidepressants + Tranquilizers	4	0.7	1	0.2	5	0.9
Anti-diabetic agents (Insulin and sulfonylureas)	10	1.7	4	0.7	14	2.4
Anti-diarrheal agents	–	–	2	0.3	2	0.3
Antiemetic	44	7.5	96	15.8	140	23.3
Anti-flatulent	–	–	2	0.3	2	0.3
Antihistamines	23	3.9	13	2.1	36	6
Antineoplastic agents	17	2.9	57	9.4	74	12.3
Antiprotozoal + Anti-leprosy agents	14	2.4	4	0.7	18	3.1
Antiviral	–	–	3	0.5	3	0.5
Appetite stimulants	–	–	5	0.8	5	0.8
Benzodiazepine antagonists	4	0.7	–	–	4	0.7
Bisphosphonates	–	–	8	1.3	8	1.3
Blood derivatives	–	–	2	0.3	2	0.3
Bronchodilators + Immunosuppressive agents + Mast cell stabilizers + Xanthine oxidase inhibitors	1	0.2	–	–	1	0.2
Cardiac drug + ACEIs*^b^ + Calcium antagonists + Cardiac glycosides	–	–	4	0.7	4	0.7
Cathartics/Laxatives	13	2.2	13	2.1	26	4.3
CNS depressants + Sedatives, hypnotics and Anxiolytics (Barbiturates and Benzodiazepines)	10	1.7	24	4	34	5.7
Coagulants and Anticoagulants	19	3.2	8	1.3	27	4.5
Colony stimulating factor (CSF)	9	1.5	5	0.8	14	2.3
Cyto-protectants (Mesna)	–	–	6	1	6	1
Devices	–	–	2	0.3	2	0.3
Dietary supplements and Vitamins	7	1.2	72	11.9	79	13.1
Diuretics	–	–	11	1.8	11	1.8
Enzymes	–	–	1	0.2	1	0.2
Expectorants	–	–	5	0.8	5	0.8
Human albumin	–	–	7	1.2	7	1.2
Immunomodulatory agents	–	–	3	0.5	3	0.5
Monoclonal antibodies	–	–	5	0.9	5	0.9
Mouth washes and gargles	12	2	–	–	12	2
Ointments/Solutions/Suspensions ophthalmic + Otic/Ophthalmic/Nasal preparations	7	1.2	4	0.7	11	1.9
Opiates	–	–	1	0.2	1	0.2
Para-sympatholytic agents	4	0.7	–	–	4	0.7
Quinolones	–	–	3	0.5	3	0.5
Replacement solutions/ORS*^c^/Minerals/Elements/Electrolytes	16	2.7	24	4	40	6.7
Skeletal muscle relaxants	4	0.7	–	–	4	0.7
Steroids	4	0.7	11	1.8	15	2.5
Sugar/Salt substitutes	2	0.3	14	2.3	16	2.6
Sympathomimetic agents	7	1.2	2	0.3	9	1.5
Thrombolytic agents	–	–	4	0.7	4	0.7
Uricosuric agents	–	–	3	0.5	3	0.5
Total	589	100	606	100	1195	

^a^ *NSAIDs* Non-steroidal anti-inflammatory drugs, ^b^ *ACEIs* Angiotensin converting enzyme inhibitors and ^c^ *ORS* Oral rehydrating salts

## Discussion

This study focused on the incidence of medication related events among cancer in-patients along with the concurrent evaluation of causes and system related factors at different levels responsible for their occurrence. Like the studies done in the past by Watts et al. [15] and Jayanti et al. [8] this study showed that the most common types of incidents reported at both sites included improper dose, monitoring errors, wrong time or schedule of administration and many other types like drug labelling, missing drug related information, dose prescribing and/or dispensing incidents. At the setting with computerized prescribing system (CPOE) the incidence of reporting any medication related event was high and that’s why most of the errors were intercepted before reaching the patients. The electronic system made it possible to analyze each medication order at several points. As compared to the electronic system the setting with hand written prescribing and electronic transcribing with only limited access to medication related data several types of incidents like labeling instructions, drug dilution and weight based dosing protocols, unnecessary medications resulting in increase in treatment cost and polypharmacy occurred frequently and many went unreported. Watts et al. showed greater incidence of similar errors including dosing errors (42%), roadmap errors (26%) and timing errors (12%) [16]. Another study by Jayanti et al. showed comparing rates of prescription error types including missing information (47.1%) and abbreviations in pre-medication (23.3%). It showed potential errors like incomplete premedication (32.7%), dosing errors in anti-cancer drugs (3%), missing dosage forms (1.2%), missing information on diluents (3.8%) and time of infusion (34.9%) [9].

Among causes the major cause was human factors resulting in the occurrence of medication related events. Human factors including performance deficit, miscalculations of dosage and/or infusion rates, behavior of HCPs were the major causes of medication related events may lead to MEs or potential ADEs. At hand written setting heavy work load and lack of staff were the major causes that resulted in errors because there is no cross check point which can analyze every order. The lack of interdisciplinary approach subjecting nursing staff to prepare medication at bed side further increases the chance of error at any setting. Electronic system at site 1 with CPOE and CDSS greatly helped out in reduction of manual workload. The medication preparation and dilution at pharmacy further reduced the chances of drug dose calculations and concentrations. As discussed by Jayanthi et al. our study also looked for the factors contributing in the occurrence of medication related incidents. Staff training in the specialty of chemotherapy and critical care, staff replacements by specialized staff for chemotherapy, supervision of residents and nursing students, improvement in the system for patient care, updating and implementing electronic system according to present requirements are the factors which will bring a great difference in the occurrence and interception of medication errors. Jayanthi et al. described similar aspects in their study like time limitations, interruptions, lack of knowledge and attention paid to drug dosing and preparation resulting in a number of errors. The limitation of both studies (at site 2 with HWP in our study) was also same as the patient profile was mostly incomplete lacking basic medical and/or medication information of the patient [11]. Watts and Parsons studied the effect of pharmacist driven intervention which showed a 50% decrease in the rate of medication errors indicating that the pharmacist evaluation greatly reduces reaching of medication error to the patient [15]. Al-Dawailie in his practice report described that a huge variety of errors were associated with hand written prescribing practice as our study reported at site 2 with HWP [17].

Our study provided a detailed overview of severity of all medication related events including medication errors without harm and the potential ADEs which are mostly not done in many studies. This study categorized outcome on the basis of medication error index presented by NCC-MERP. Our study showed that at the setting working on CPOE/CDSS electronic system most of the incidents are intercepted before reaching the patient. Many errors which reached the patients were intercepted by the pharmacist’s rounds along with medical practitioners at each unit. During ward rounds and during medication review electronically medical interventions were done and discussed with the practitioners which if approved was applied. While at the setting with a limited access to drug databases a lot of incidents reached the patients without interception. A large number of incidents reached the patients which needed evaluation for harm. This lack of updated system and least involvement of clinical pharmacist during ward rounds and during dispensing from pharmacies reduced the chances of medical interventions which could lead to permanent patient harm, increasing length of stay, and medication cost. Ranchon et al. provided almost similar results on the severity of outcomes. The study suggests that the toxic nature of cytotoxic drugs, patient condition and comorbidities, complex regimens and frequent dose adjustments and calculations makes the outcomes severe. The outcome evaluation though requires close observation which may affect the results of such studies.

The errors were highly reported at the level of prescription at prescriber’s office and oncology ward. Error discovery was higher at pharmacy level at site 1 working on electronic system with CPOE and CDSS as compared to site 2 with most of the work done manually and least updated electronic system. It elucidates that pharmacy department is efficiently involved in the medication utilization review process at site 1 with complete access to patient medication and medical record based electronic system which makes it easier to detect any medication related event as compared to site 2. The pharmacist’s involvement during medication use process greatly reduces the occurrence of medication errors in chemotherapy patients. Ranchon et.al showed an incidence of 5.2% prescription errors in cancer patients which is quite low as compared to the rate at site 2 in our study and almost equal to site 1. The hospital reported by Ranchon et al. had a same electronic system as at our study’s site 2 and a handwritten prescription approach but the prescriptions were sent to pharmacy department before administering to the patients for complete review which greatly reduced the number of medication errors reaching the patient. The study showed a low percentage of administration (0.02%) and dispensing (0.16%) errors as compared to our study. Both; our study and the Ranchon et al. study; has under-reported errors at administration and pharmacy level due to blame and shame approach and lack of time and observation strategies. Watts et al. showed similar results while studying chemotherapy medication errors at a pediatric hospital at a low rate of 6/1000 patient visits while 3.9/1000 dispensing orders. After implementation of a pharmacy surveillance system the error rate was further reduced by 50% at both ends. The study focused on the need of multidisciplinary review of policies and procedures to ensure patient safety [15].

Our study found a higher incidence of medication related events (potential ADEs) at site 2 (HWP system) while rate of medication errors without harm and interventions was reported highly at site 1. The higher incidence of potential ADEs at site 2 might be due to the absence of clinical pharmacists, lack of staff training, less updated software and communication among HCPs. Errors nature was preventable mostly concluding that proper cross checking could prevent a greater incidence before reaching the patient.

Drugs highly at risk of medication errors in our study were antibiotics the most commonly prescribed class of drugs in our health care settings, anti- analgesics and antipyretics supplements, antacids, anti-cancer drugs, and electrolytes because of less consideration while prescribing these classes of drugs. Tang et al. found similar results in his study. Antibiotics were the leading class of drugs (38.9%) subjected to errors due to its prescribing pattern and confusion with other drugs having similar names. Other drugs reported were analgesics (6.9%), electrolytes (8.4%), antidiabetics (6.9%) and other classes (38.9%). Ranchon et al. predictors of prescribing errors among cancer patients showed a 3.15% error rate among all antineoplastic prescriptions [13].

A detailed study by Richard J. Fitz Gerald showed the importance of accurate medication and medical history in the prevention of medication errors. Errors were more common when patients admitted at hospitals because of incomplete medication histories. Many drugs can cause or mask many conditions like preventing tachycardia by beta adrenoceptor antagonists in hemorrhagic patients, abdominal pain by corticosteroids in perforated duodenal ulcers and/or masking the effects of investigations as thyroid function tests by amiodarone. It also showed that to prevent such errors pharmacists can perform much better roles as compared to physicians and nurses due to their ability to take detailed medication histories and compliance records [16].

## Conclusion

Our study revealed many issues of concern at cancer care settings working on different systems. CPOE with CDSS will greatly reduce the occurrence of medication errors by decreasing the workload of every member involved in pharmaceutical care. Regular staff training and incorporation of pharmacist at different checkpoints will improve health care provision. Regular root cause analysis by a health care team will greatly improve the system making better utilization of resources. The limitation of this study was the less number of data collection members. The handwritten prescriptions, lack of ward pharmacists, under-reporting of mediation errors and time constraints at site 2 may affect the actual number of errors.

### Study strengths

This study has focused on the root causes and contributing factors in two systems working on different approaches of prescribing, dispensing and administration. The incidence and prevalence of medication related incidents are mostly studied without considering the factors involved which are leading to their occurrence. For improvement in our health system and patient safety more studies like this are needed to be done on a broader level.

### Limitations

The limitations of this study includes

- Time limit as the study was done only for few months. It is needed to be done for a period of a year at least to better understand the occurrence rates of medication related events.
- The sample size needed to be increased which was limited because of lack of data collection personnel.
- All the health care team members including prescribers, pharmacists and nurses are need to be a part of such research team to avoid any biasness and misinterpreting any medication order.
- Many of the events may go un-reported due to lack of personnel.
- A more strong statistical analysis is needed for handling such a huge data with so many variables.

## Data Availability

The datasets used and/or analyzed during the current study are available from the corresponding author on reasonable request.

## Abbreviations

ADEs: Adverse drug events
BNF: British National Formulary
CDSS: Computerized decision support system
CI: Confidence interval
CPOE: Computerized Prescription Order Entry
HCPs: Health care professionals
HWP: Hand written prescription
MEs: Medication errors
NCC-MERP: National Coordinating Center for Medication Error Reporting and Prevention
NCCN: National Comprehensive Cancer Network
